# Mitochondrial DNA haplogroups confer differences in risk for age-related macular degeneration: a case control study

**DOI:** 10.1186/1471-2350-14-4

**Published:** 2013-01-09

**Authors:** M Cristina Kenney, Dieter Hertzog, Garrick Chak, Shari R Atilano, Nikan Khatibi, Kyaw Soe, Andrew Nobe, Elizabeth Yang, Marilyn Chwa, Feilin Zhu, Masood Memarzadeh, Jacqueline King, Jonathan Langberg, Kent Small, Anthony B Nesburn, David S Boyer, Nitin Udar

**Affiliations:** 1 Gavin Herbert Eye Institute, Univeresity of California Irvine, Hewitt Hall, Room 2028, 843 Health Science Rd, Irvine, CA 92697, USA; 2 Loma Linda University School of Medicine, Loma Linda, CA, USA; 3 Northwestern Feinberg School of Medicine, Chicago, IL, USA; 4 Cedars-Sinai Medical Center, Los Angeles, CA, USA; 5 Retina-Vitreous Associates Medical Group, Beverly Hills, CA, USA

**Keywords:** Age-related macular degeneration, Mitochondrial haplogroups, mtDNA, CFH, ARMS2

## Abstract

**Background:**

Age-related macular degeneration (AMD) is the leading cause of vision loss in elderly, Caucasian populations. There is strong evidence that mitochondrial dysfunction and oxidative stress play a role in the cell death found in AMD retinas. The purpose of this study was to examine the association of the Caucasian mitochondrial JTU haplogroup cluster with AMD. We also assessed for gender bias and additive risk with known high risk nuclear gene SNPs, ARMS2/LOC387715 (G > T; Ala69Ser, rs10490924) and CFH (T > C; Try402His, rs1061170).

**Methods:**

Total DNA was isolated from 162 AMD subjects and 164 age-matched control subjects located in Los Angeles, California, USA. Polymerase chain reaction (PCR) and restriction enzyme digestion were used to identify the J, U, T, and H mitochondrial haplogroups and the ARMS2-rs10490924 and CFH-rs1061170 SNPs. PCR amplified products were sequenced to verify the nucleotide substitutions for the haplogroups and ARMS2 gene.

**Results:**

The JTU haplogroup cluster occurred in 34% (55/162) of AMD subjects versus 15% (24/164) of normal (OR = 2.99; p = 0.0001). This association was slightly greater in males (OR = 3.98, p = 0.005) than the female population (OR = 3.02, p = 0.001). Assuming a dominant effect, the risk alleles for the ARMS2 (rs10490924; p = 0.00001) and CFH (rs1061170; p = 0.027) SNPs were significantly associated with total AMD populations. We found there was no additive risk for the ARMS2 (rs10490924) or CFH (rs1061170) SNPs on the JTU haplogroup background.

**Conclusions:**

There is a strong association of the JTU haplogroup cluster with AMD. In our Southern California population, the ARMS2 (rs10490924) and CFH (rs1061170) genes were significantly but independently associated with AMD. SNPs defining the JTU mitochondrial haplogroup cluster may change the retinal bioenergetics and play a significant role in the pathogenesis of AMD.

## Background

Age-related macular degeneration (AMD) is the leading cause of blindness among the elderly population in the developed world and it is anticipated that its prevalence will rise. Risk factors for AMD include Caucasian race, smoking and family history. Clinical characteristics of early AMD are subretinal drusen and loss of retinal pigment epithelium (RPE). Late AMD exists in two forms: dry AMD (atrophic, stage 4) which has progressive loss of RPE cells and overlying photoreceptors and wet AMD (neovascular, stage 5) which makes up approximately 15% of the cases and is characterized by choroidal neovascularization and disciform scar formation. Both forms can result in severe central vision loss.

Mitochondria are critical organelles that provide energy to the cell via oxidative phosphorylation (OXPHOS). Mitochondria are unique in that they have their own DNA (mtDNA) which is highly polymorphic with 16,569 nucleotide pairs that code for 37 genes including 13 OXPHOS protein subunits, 2 ribosomal RNAs and 22 transfer RNAs [1,2]. The mtDNA lacks histones and has poor DNA repair systems so it is at greater risk of damage compared to nuclear DNA. Human retinal cells are very metabolically active and have evidence of oxidative damage in the retinal mtDNA, including high degrees of pathologic heteroplasmy, large deletions and nucleotide substitutions. Recently, studies have shown that aging and AMD retinas have increased mitochondrial structural abnormalities and elevated levels of mtDNA damage [3-7].

Another mechanism by which diseases can occur is through the association of mtDNA haplogroups which represent different ethnic populations of the world. A specific haplogroup is defined by variations in mtDNA sequences within the maternal lineages that have accumulated over thousands of years and represent the geographic origin of that population. The oldest haplogroups (L1-L3) originated from Africa (130 K-170 K years) while the most recent haplogroups (A, B, C, D, X) originated within the North and South American continents (18 K-34 K years) (http://www.mitomap.org). The European haplogroups (H, I, J, K, T, U, V, W and X) are approximately 30 K-50 K years old. It is likely that the haplogroup single nucleotide polymorphism (SNP) variants may have functional consequences. Since mitochondria are critical for energy production, the haplogroup-related SNPs may be related to partial uncoupling of OXPHOS and decreased efficiency of ATP production [8-10]. This means that each haplogroup, with its different set of SNPs, can have unique bioenergetic properties and responses to oxidative stressors. The haplogroup defining SNPs may modify the required mitochondrial energetics of that population to meet the needs of their environment [11] and therefore may have varying biological effects on the cells.

Studies have shown that the age-related diseases, such as Alzheimer’s and Parkinson’s, are associated with specific haplogroups [12-15]. Large soft drusen, retinal pigment abnormalities and the wet forms of AMD, an age-related eye disease, have also been shown to be associated with some European haplogroups [16-20]. Correlations between other ocular diseases and haplogroups are also being reported. Susceptibility to pseudoexfoliation glaucoma is decreased in patients with a U haplogroup but increased with T or L2 haplogroups [21,22]. In a Saudi Arabian population, there is an increased risk of primary open-angle glaucoma in patients with the African L haplogroups, excluding L2 haplogroup [23]. In addition, there is a higher prevalence of diabetic retinopathy in type 2 diabetic patients with the mtDNA T haplogroup background [24].

There are two major susceptibility genes associated with AMD in certain populations. The CFH gene polymorphism (rs1061170), T1277C (Tyr402His) has been associated with the development and progression of AMD [25-29] in Caucasian populations but not Asians [30-32]. The CFH protein blocks C3 to C3b activation, causes C3b degradation, and thereby regulates the alternative complement pathway. Both aging and smoking can decrease the CFH plasma levels [33] and which can lead to increased inflammation [34,35]. The ARMS2/LOC387715 gene polymorphism (rs10490924) is a missense SNP transversion from G > T (Ala69Ser). In a North American population, TT homozygosity is associated with the wet and dry forms of advanced AMD, showing an allele-dose effect [36]. Studies based on Japanese AMD populations have found that the SNP (rs10490924) in the LOC387715 gene is associated with the wet form of AMD, [37-39] which has been confirmed in both American [40-42] and Indian populations [43]. Fritsche and coworkers expressed the LOC387715 mRNA [44] and reported a mitochondrial association [45] although this has not been found by others [46]. Some investigators suggest that the ARMS2 gene codes for a secreted protein that binds to extracellular matrix [47]. Baas and coworkers have shown significant association for three SNPs of the glucose transporter gene (SLC2A1) in a single cohort, but when applied to additional study populations, the results showed an inconsistent, non-significant association [48]. Based upon these findings, they suggest that across populations there is heterogeneity of AMD risk factors which exists as the rule rather than the exception.

It has already been shown that the clinical phenotypes of diseases can be influenced through synergistic effects of nuclear genes with the mitochondrial genome [49,50]. For example, Leber’s hereditary optic neuropathy (LHON) individuals harboring the milder mutations at positions 11778, 14484, and 10663 have increased severity and probability of blindness if they have a J haplogroup background [49,51]. The LHON patients with the 3460 mutation on a Uk mtDNA haplogroup background were higher risk for vision loss [52] while the H haplogroup protected from the disease [53]. In contrast, the J haplogroup background in HIV infected patients protects them against progression of neuroretinal disorder (NRD) [54]. The present study was designed to assess the frequency of the JTU haplogroup cluster in our AMD population and examine the potential additive associations of the ARMS2-rs10490924 and CFH-rs1061170 risk alleles.

## Methods

### AMD classification

The subjects underwent a complete dilated ophthalmic examination by Board certified ophthalmologists (D.S.B., A.B.N., K.S., M.C.K.) including both slit lamp examination and an indirect ophthalmic exam with a 90 diopter lens or a fundus contact lens. Fundus photos, fluorescence and/or indocyanine green angiography were performed. The photos and angiograms were read by masked graders who were board certified retinal specialists [29]. Subjects were graded according to the Clinical Age-Related Maculopathy Staging System (CARMS) [55]. Grade 3 had large soft drusen or several intermediate size drusen or drusenoid retinal pigment epithelial detachments and for this study is referred to as Early AMD. In this study the term Late AMD is the combination of Grade 4 which is geographic atrophy and grade 5 which is neovascular or serous exudative AMD. No stage 1 or 2 AMD patients were included in this study.

### Data collection

Institutional review board approval was obtained from the University of California in Irvine (#2003-3131) and from Cedars-Sinai Medical Center (#1708) with prior patient consent. This study examined DNA samples obtained from 162 AMD patients and 164 age-matched controls (Table 1). The patients with AMD were mainly from a private practice retina specialist office (Retina-Vitreous Associates Medical Group) and the controls were from a private general ophthalmology office (American Eye Institute). The offices are approximately 2 miles apart and serve the same demographic and ethnic populations, which is predominantly European haplogroup Caucasian. There was no statistical difference in the ages between the case and control populations (78.03 ± 0.68 versus 76.4 ± 0.56, p = 0.06). When analyzed according to gender, the mean ages were not significantly different (female-AMD, 78.29 ± 0.85 versus female-control, 76.61 ± 0.72, p = 0.14; and male-AMD, 77.32 ± 1.17 versus male-control, 76.1 ± 0.90, p = 0.40). In the AMD population there were twice as many females as males (109 versus 53) but the mean ages were similar (female-AMD 78.29 ± 0.85 and male-AMD, 77.32 ± 1.17, p = 0.72). The control population had 94 females to 70 males with similar ages (female-control 76.61 ± 0.72 and male-control, 76.10 ± 0.90, p = 0.65). 10 ml sample of peripheral blood was collected in tubes containing 10 mM EDTA. DNA extraction was performed with a DNA extraction kit (PUREGENE, Minneapolis, MN).

**Table 1:** Demographics of AMD and normal subjects

	**Total AMD**	**Normal**	**p-value**
**n**	162	164	
**Median Age**	79	75	
**Mean Age ± SEM**	78.03 ±0.68	76.4 ±0.56	0.06
**Female (n)**	109	94	
**Median Age**	79	75	
**Mean Age ± SEM**	78.29 ±0.85	76.61 ±0.72	0.14
**Male (n)**	53	70	
**Median Age**	78	75	
**Mean Age ± SEM**	77.32 ±1.17	76.1 ±0.90	0.40

*SEM*, standard error of the mean; *n*, number of samples.

### Mitochondrial haplogroup analyses

**Figure 1:**
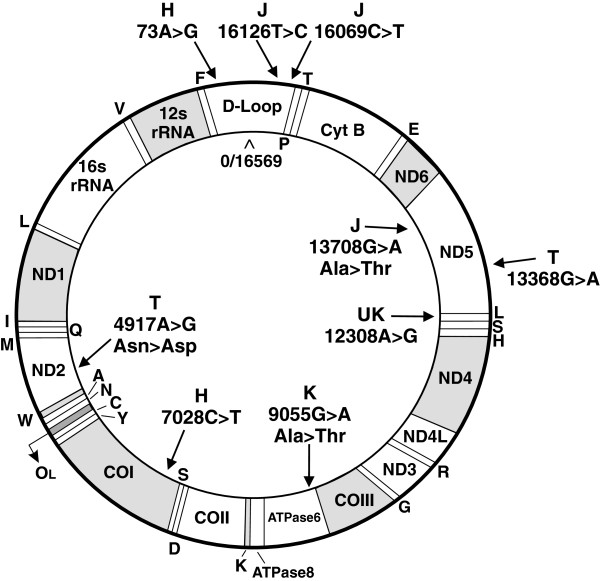
**Schematic of circular mtDNA showing SNPs that define the H, J, T and U haplogroups. **Cyt B, Cytochrome B; ND, NADH dehydrogenase; CO, Cytochrome *c* oxidase; ATPase, ATP synthase; D-loop, non-coding region between 16024 and 576.

Samples were analyzed using PCR, restriction enzyme digestion and sequencing of the mtDNA to identify the mitochondrial haplogroup of each patient as described previously [18]. The SNPs defining the J haplogroup were G13708A, C16069T and T16126C (Figure 1). The T defining SNPs were A4917G and G13368A. The U defining SNPs were positive for A12308G but negative for G9055A. The H defining SNPs were T7028C and G73A.

### DNA amplification

Polymerase Chain Reaction (PCR) was used to amplify desired DNA regions from 100 ng of DNA. The PCR reaction for the region flanking the G > T SNP of rs10490924 in the ARMS2 was performed with an annealing temperature of 60°C (forward primer sequence 5^′^GCACCTTTGTCACCACATT3^′^ and reverse primer sequence 5^′^GCCTGATCATCTGCATTTCT3^′^). The primers and PCR conditions for the CFH-rs1061170 gene were described previously [29].

### Restriction digestion

**Figure 2:**
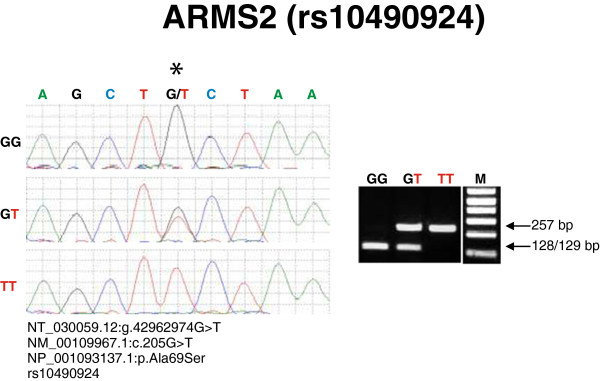
**Sequencing and restriction enzyme digestion of the ARMS2-rs10490924 SNP.** Panel: Left – Sequence chromatogram of the GG homozygote, GT heterozygote and TT homozygote of the ARMS2-rs10490924 SNP. Right - 1.5% agarose gel showing the single lower band representing GG and the single higher band being TT. The double band represents heterozygous GT after PCR and digestion with PvuII. M = marker.

Restriction endonuclease digestion was performed following PCR amplification. Restriction enzymes were used to digest PCR products, according to the manufacturer’s recommended protocol (New England Biolabs, Ipswich, MA). Digested samples were separated by electrophoresis on 1.5% agarose gels and stained with ethidium bromide. Genotyping according to DNA fragment size following digestion and electrophoresis is as follows:

For ARMS2-rs10490924 SNP (product size 257 bp), PvuII will digest the wild type G allele into fragments of 129 and 128 bp, while an allele with the G > T SNP does not cut and appears as a band at 257 bp (Figure 2). Accordingly, bands appear at 257, 129, and 128 bp in a GT heterozygote.

The PCR product for CFH-rs1061170 SNP was digested with NlaIII restriction enzyme as described previously [29]. The uncut product is 469 bp and the length of the restriction fragments for the T allele are 6, 74, 89 and 300 bp while the C allele appears as 6, 74, 85, 89, and 215 bp (data not shown).

### Sequencing

Once genotypes were assigned by means of restriction digest, samples of PCR product were taken from each of the alleles for the ARMS2-rs10490924 SNP in order to confirm the validity of the genotypes as determined by the digests. These samples were treated with ExoSAP-IT (USB Corp. Cleveland, OH) according to the manufacturer’s protocol. The samples were then sequenced at the UCLA Sequencing and Genotyping Core, Los Angeles, CA. All sequenced samples matched the genotypes obtained by restriction digest.

### Statistical analysis

Statistical analysis was performed using Simple Interactive Statistical Analysis (SISA) internet software (Quantitative Skills, The Netherlands) and the Fisher’s exact test using GraphPad Prism software (San Diego, CA).

## Results

A cohort of 162 case and 164 control subjects were genotyped for individual Caucasian mitochondrial haplogroups J, T, U and H. Genotypes were determined according to the specified migration pattern of the digested products and the data were used to assign the representative haplogroups (Table 2). Statistical analyses were carried out for the occurrence of each haplogroup within the case and control populations. The occurrence of the common Caucasian H haplogroup was similar in the case (31%, 51/162) and control populations (30%, 50/164, p = 0.94, Table 2). In contrast, the northern European/Caucasian haplogroup JTU cluster was 34% (55/162) of the total AMD population but only 15% (24/164) of the age-matched normal population (OR = 2.99; 95% CI = 1.74-5.15; p = 0.0001). These findings show that AMD is more commonly found in subjects with the JTU mitochondrial haplogroup cluster than in subjects with the H haplogroup. We then segregated the samples based on their gender and analyzed the distribution. The JTU cluster was slightly higher for AMD in males compared to controls (OR = 3.98, p = 0.005) than females versus controls (OR = 3.02, p = 0.001) but this may be related to the smaller numbers of males in the case population.

**Table 2:** mtDNA cluster haplogroups and gender

	**AMD**	**Normal**	**OR**	**95%CI**	**p-value**
**TOTAL**					
H	51/162 (31%)	50/164 (30%)	1.05	0.66-1.67	0.94
JTU cluster	55/162 (34%)	24/164 (15%)	2.99	1.74-5.15	0.0001
**FEMALE**					
H	35/109 (32%)	30/94 (32%)	1.00	0.56-1.82	0.90
JTU cluster	37/109 (34%)	16/94 (17%)	3.02	1.56-5.85	0.001
**MALE**					
H	16/53 (30%)	20/70 (29%)	1.08	0.49-2.37	0.99
JTU cluster	18/53 (34%)	8/70 (11%)	3.98	1.57-10.10	0.005

*OR*, Odds Ratio; *CI*, Confidence Interval.

In the total (early + late) AMD population, the ARMS2-rs10490924 SNP, was represented in a frequency of allele distribution of 45% high risk T allele while the normal population was 28% T allele (p < 0.00001, Table 3). The early AMD subset had 42% T allele frequency (p = 0.007) and the late AMD subset had 48% T distribution (p < 0.00001) compared to the 28% T allele in the control group. The allele distribution for the CFH SNP was 42% high risk C allele in the total AMD group compared to 29% in the normal populations (p = 0.0006), while the late AMD population had 44% C allele distribution (p = 0.0.0006). In the early AMD populations the C risk allele distribution was 39% (p = 0.06).

**Table 3:** Frequency of alleles in AMD and normal populations

	**Early**	**Late**	**Total AMD**	**Normal**
**ARMS2 Allele T (risk)**	50/120	96/202	146/322	91/328
	(42%)	(48%)	(45%)	(28%)
**ARMS2 Allele G**	70/120	106/202	176/322	237/328
	(58%)	(52%)	(55%)	(72%)
**CFH Allele C (risk)**	43/110	83/188	126/298	94/326
	(39%)	(44%)	(42%)	(29%)
**CFH Allele T**	67/110	105/188	172/298	232/326
	(61%)	(56%)	(58%)	(71%)

The dominant odds ratios (OR) were calculated by determining the prevalence of the risk allele (heterozygous + homozygous risk alleles) compared to normal subjects with no copy of the risk allele (homozygous wildtype; Table 4). Assuming a dominant effect, the T risk allele for the ARMS2-rs10490924 SNP was significantly associated with AMD (OR = 4.72; p = 0.00001). This high risk allele was also associated in the late AMD (OR = 3.13, p = 0.0001) but not the early AMD group (OR = 1.81; p = 0.07). With respect to C risk allele of the CFH-rs1061170 SNP, assuming a dominant risk, the OR was 1.71 for the total AMD population compared with the control population (p = 0.027). The increased association for the C allele was also found in this late AMD population (OR = 2.05; p = 0.014). This demonstrates that our case population had ARMS2-rs10490924 and CFH-rs1061170 high risk alleles associated with AMD which is similar to results found in the literature [56].

**Table 4:** Genotypes and odds ratios in age-related macular degeneration (AMD) and normal patients with risk allele, assuming a dominant effect

	**Early AMD**	**Late AMD**	**Total AMD**	**Normal**
**ARMS2 Homozygous T (risk)**	11/60 (18%)	19/101 (19%)	30/161 (19%)	10/164 (6%)
**ARMS2 Homozygous G**	21/60 (35%)	24/101 (24%)	45/161 (28%)	83/164 (51%)
**ARMS2 Heterozygous**	28/60 (47%)	58/101 (57%)	86/161 (53%)	71/164 (43%)
**OR (p-value)**	1.81 (0.07)	3.13 (0.0001)	4.72 (0.00001)	
**CFH Homozygous C (risk)**	11/55 (20%)	18/94 (19%)	29/97 (20%)	16/163 (9.8%)
**CFH Homozygous T**	23/55 (42%)	29/94 (31%)	52/97 (35%)	85/163 (52%)
**CFH Heterozygous**	21/55 (38%)	47/94 (50%)	68/97 (45%)	62/163 (38%)
**OR (p-value)**	1.27 (0.54)	2.05 (0.014)	1.71 (0.027)	

*OR*, Odds Ratio.

We then examined the association of the high risk nuclear alleles for subjects that had mitochondrial backgrounds of either H haplogroup or the JTU haplogroup for the case and control populations (Table 5). For the ARMS2 gene, we found 18% of the AMD subjects had the homozygous T with the H haplogroup (9/50) while 18% had the JTU cluster haplogroup (10/55). The homozygous high risk C allele of the CFH gene was associated with 23% of the H haplogroup (11/47) and 18% of the JTU cluster haplogroup (9/51). Then we analyzed the likelihood of increased risk for the ARMS2-rs10490924 or CFH-rs1061170 SNPs on the JTU cluster background versus the H background in AMD subjects (Table 6). There were weak odds ratios and no significant differences when we compared the H and JTU cluster backgrounds, indicating that having a JTU background may not have additive risk for the ARMS2-rs10490924 or CFH-rs1061170 SNPs when compared to the H haplogroup background. However, additional studies using larger populations are needed to investigate the interactions between risk factors and “mtDNA defined” genetic backgrounds since the power of our study is low.

**Table 5:** Genotypes found in AMD subjects and normal subjects with either the H haplogroup background or JTU haplogroup background

**Haplogroup and ARMS2 (G > T)**			
	Homozygous T	Homozygous G	Heterozygous
**AMD**			
**H**	9/50 (18%)	11/50 (22%)	30/50 (60%)
**JTU cluster**	10/55 (18%)	17/55 (31%)	28/55 (51%)
**Normal**			
**H**	5/52 (10%)	23/52 (44%)	24/52 (46%)
**JTU cluster**	0/24 (0%)	11/24 (46%)	13/24 (54%)
**Haplogroup and CFH (T > C)**			
	Homozygous C	Homozygous T	Heterozygous
**AMD**			
**H**	11/47 (23%)	14/47 (30%)	22/47 (47%)
**JTU cluster**	9/51 (18%)	18/51 (35%)	24/51 (47%)
**Normal**			
**H**	6/51 (12%)	24/51 (47%)	21/51 (41%)
**JTU cluster**	1/24 (4%)	12/24 (50%)	11/24 (46%)

**Table 6:** Odds ratio of risk alleles in AMD population on a JTU haplogroup background versus H background

	**Homozygous Risk Allele OR; (p-value); 95%CI**	**Homozygous Wildtype Allele OR; (p-value); 95%CI**	**Heterozygous OR; (p-value); 95%CI**
**ARMS2**			
**JTU Cluster vs H**	0.51; (0.28) 0.185 – 1.387	1.845; (0.25) 0.766-4.439	1.01; (0.86) 0.457 – 2.235
**CFH**			
**JTU Cluster vs H**	0.326; (0.529) 0.037 – 2.87	1.125; (0.99) 0.426 – 2.97	1.21; (0.89) 0.455 – 3.213

*OR*, Odds Ratio; *CI*, Confidence Interval.

## Discussion

Increasing evidence shows that mitochondrial dysfunction plays a role in development and progression of AMD. Haplogroups are defined by an accumulation of SNPs that have over thousands of years become representative of that specific geographic population. The most common Caucasian European haplogroups is H and it is the basis for the Cambridge reference sequence by MitoMap (http://www.MitoMap.org). The J, T, and U haplogroups have their own defining SNPs, some of which are non-synonymous (amino acid changing) and others which occur in the non-coding MT-Dloop, a region critical for replication and transcription. Different SNP variants may change retinal bioenergetics and energy production levels causing a) decreased OXPHOS efficiencies; b) increased ROS production; c) elevated oxidative stress and apoptosis and d) elevated levels of cell death, which may contribute to AMD and other retinal diseases. It is well recognized that oxidative stress is associated closely with aging and age-related diseases. Therefore, it must be noted that while investigating the mtDNA variants that define the haplogroups, it is important to assess the ages of the case and control populations because minor age differences may lead to false positive associations.

The present study showed the mitochondrial haplogroup cluster JTU was significantly associated with the development of AMD (p = 0.0001) while H, the most common Caucasian haplogroup, had no risk association with the disease. Our results support previous findings that the haplogroup T-associated SNP A4917G is an independent predictor of AMD [17] and two variants of the T2 haplogroup, A11812G of MT-ND4 and A14233G of MT-ND6, are 2.5 times more likely to be associated with advanced AMD than the age-matched control subjects [19]. Analyses of Middle European Caucasians showed that the haplogroup J was associated with wet AMD while the H haplogroup was protective [20]. In an Australian population, the early AMD signs of large soft drusen and retinal pigment abnormalities have been associated with J and U haplogroups [16]. The OXPHOS “uncoupling” of mtDNA associated with the J, T and U haplogroups are more commonly found in populations that originated in Northern European colder climates and these are the ones that are often associated with altered risk in the aging-related disorders of Parkinson’s disease and Alzheimer’s disease, and now AMD. Recent studies have shown that haplogroup J cybrids (cytoplasmic hybrids) and Uk cybrids differ in the mtDNA content, levels of ATP production and OXPHOS capacity compared to H haplogroup cybrids [57,58]. This has led to speculation that mtDNA variants that define haplogroups might mediate cellular signaling pathways and influence the susceptibility to different diseases.

Upon recalculation of the risks based on gender, we also found a slightly higher Odds Ratio associated with males as compared to females but further analyses with larger numbers of subjects are needed to definitively make this association. It is possible that nuclear modifier elements may influence gender bias associated with aging and/or diseases. One study showed that cells with the J haplogroup backgrounds can increase mtDNA copy numbers more rapidly than the H haplogroups [59]. It has been proposed that the SNPs that define the JTU clusters may partially “uncouple” OXPHOS and alter the mitochondrial energy production efficiency [8]. Sperm with the T and U haplogroups showed lower motility than the sperm with the common European haplogroup H [60]. This type of “uncoupled” change means that more calories would be consumed for the same amount of ATP produced and as a result, the mitochondrial ROS levels would be higher [9,10]. These elevated ROS levels could in turn lead to higher levels of oxidative damage to DNA, lipids and proteins. As the retina is one of the most metabolically active tissues in the body, even a partial decline in the energy production efficiency might significantly affect the retinal function.

Rivera et al. implicated the G > T (Ala69Ser) SNP in exon 1 of LOC387715 (ARMS2-rs10490924) as a possible susceptibility candidate for AMD, accounting for linkage to the 10q26 region [61]. Shortly thereafter, Schmidt et al., conducted a study that also identified the G > T polymorphism in ARMS2-rs10490924 as an AMD-susceptibility allele [62]. Further studies revealed a strong association between AMD populations and the number of G > T alleles at ARMS2-rs10490924 [63,64]. Assuming a dominant effect, we found that the ARMS2-rs10490924 risk allele is associated with the late form of AMD but not the early form. Our findings agree with Ross and coworkers who also showed LOC387715 associated with the more advanced clinical-based cases but not the early AMD cases [65]. It is also consistent with two other studies that show that the presence of the T risk allele for the LOC387715 gene is associated with the more severe, wet form of AMD [39,41]. From our case–control study, we found that the association between the T allele for the ARMS2 gene (rs10490924) and AMD is independent of the patient’s mtDNA haplogroup background. However, our population numbers are relatively small and our results need to be corroborated with a larger size population.

Our results show strong evidence for the CFH risk allele C (rs1061170) to be a susceptibility allele for AMD in our population. As the allele T is more common in the control population, it indicates that tyrosine at residue 402 may have a protective function. In addition to the compelling CFH genetic studies, there is additional biological evidence, such as localization of complement protein in drusen deposits in AMD patients that implicate inflammation in the pathogenesis of AMD. The association of AMD with nuclear high-risk genes varies among the European, Japanese, Chinese and other ethnic populations [30,66-68]. As ethnic variations can be identified through the evolutionary SNPs that categorize a variety of ancestral mitochondrial haplogroups and migration patterns, [9,10,69,70] we hypothesized that SNP variations of mtDNA may interact differently with the high risk nuclear genes in AMD. We analyzed both the high risk nuclear genes and the Northern European JTU haplogroup cluster with respect to AMD. Our data shows that the mitochondrial JTU haplogroup cluster was an independent risk factor for AMD and not additive to the risk alleles of ARMS2-rs10490924 and/or CFH-rs1061170 SNPs. However, future studies with larger populations will need to be conducted to determine if the individual J, T or U haplogroups have additive risk to the nuclear risk genes or environmental factors such as smoking and obesity [62,64].

## Conclusions

Age-related macular degeneration (AMD) is the major cause of vision loss in the elderly, Caucasian population. In this study, analyses of 326 individuals showed that the JTU haplogroup cluster occurred in 34% of AMD subjects versus 15% of normal subjects (OR = 2.99; p = 0.001). This association was slightly greater in males (OR = 3.98, p = 0.005) than the female populations (OR = 3.02, p = 0.001). Assuming a dominant effect, the risk alleles of two known nuclear genes, ARMS2 (G > T; Ala69Ser, rs10490924) and CFH (T > C; Try402His, rs1061170), were significantly associated with the AMD populations (p = 0.0001 and p = 0.0.027). However, we found there was no additive risk for the ARMS2 or CFH SNPs on the JTU haplogroup backgrounds. Our findings are significant because this data suggests that (1) both nuclear and mitochondrial genomes are significantly but independently associated with AMD, and (2) the Northern European ancestral mtDNA sequence variants may have SNPs that contribute to altered mitochondrial efficiency associated with AMD.

## Abbreviations

AMD: Age-related macular degeneration; ARMS2: Age-related maculopathy susceptibility 2; CFH: Complement factor H; PCR: Polymerase chain reaction; mtDNA: Mitochondrial DNA; nDNA: Nuclear DNA; SNP: Single nucleotide polymorphism; OXPHOS: Oxidative phosphorylation.

## Competing interests

The authors declare they have no competing interests. This research was conducted in accordance with institutional review board approved procedures.

## Authors’ contributions

MCK helped to design the study, interpret data, performed statistical analyses, and write the manuscript. DH, GC, NK, KS, EY, MC, FZ, MM, JK, JL and AN participated in mtDNA haplogroup identification by PCR and restriction enzyme digestion and analyses of the CFH and ARMS2 genotypes. SRA performed PCR and sequencing analyses. KS performed clinical examinations on the patients and provided blood samples. ABN performed clinical examinations on the patients and provided blood samples. DSB performed clinical examinations on the patients and provided blood samples. NU designed primers, performed statistical analyses, helped to conceive the study and write the manuscript. All authors read and approved the final manuscript.

## Pre-publication history

The pre-publication history for this paper can be accessed here:

http://www.biomedcentral.com/1471-2350/14/4/prepub
